# Evaluation of PCR on Bronchoalveolar Lavage Fluid for Diagnosis of Invasive Aspergillosis: A Bivariate Metaanalysis and Systematic Review

**DOI:** 10.1371/journal.pone.0028467

**Published:** 2011-12-02

**Authors:** Wenkui Sun, Ke Wang, Wei Gao, Xin Su, Qian Qian, Xin Lu, Yong Song, Yaling Guo, Yi Shi

**Affiliations:** 1 Department of Respiratory Medicine, Jinling Hospital, Clinical School of Nanjing, Second Military Medical University, Nanjing, China; 2 Institute of Respiratory Diseases, First Affiliated Hospital, Guangxi Medical University, Nanning, China; 3 Department of Respiratory Medicine, Jinling Hospital, Nanjing University School of Medicine, Nanjing, China; 4 First Department of Respiratory Medicine, Nanjing Chest Hospital, Nanjing, China; 5 Department of Respiratory Diseases, Nursing School, Guangxi Medical University, Nanning, China; New York State Health Department and University at Albany, United States of America

## Abstract

**Background:**

Nucleic acid detection by polymerase chain reaction (PCR) is emerging as a sensitive and rapid diagnostic tool. PCR assays on serum have the potential to be a practical diagnostic tool. However, PCR on bronchoalveolar lavage fluid (BALF) has not been well established. We performed a systematic review of published studies to evaluate the diagnostic accuracy of PCR assays on BALF for invasive aspergillosis (IA).

**Methods:**

Relevant published studies were shortlisted to evaluate the quality of their methodologies. A bivariate regression approach was used to calculate pooled values of the method sensitivity, specificity, and positive and negative likelihood ratios. Hierarchical summary receiver operating characteristic curves were used to summarize overall performance. We calculated the post-test probability to evaluate clinical usefulness. Potential heterogeneity among studies was explored by subgroup analyses.

**Results:**

Seventeen studies comprising 1191 at-risk patients were selected. The summary estimates of the BALF-PCR assay for proven and probable IA were as follows: sensitivity, 0.91 (95% confidence interval (CI), 0.79–0.96); specificity, 0.92 (95% CI, 0.87–0.96); positive likelihood ratio, 11.90 (95% CI, 6.80–20.80); and negative likelihood ratio, 0.10 (95% CI, 0.04–0.24). Subgroup analyses showed that the performance of the PCR assay was influenced by PCR assay methodology, primer design and the methods of cell wall disruption and DNA extraction.

**Conclusions:**

PCR assay on BALF is highly accurate for diagnosing IA in immunocompromised patients and is likely to be a useful diagnostic tool. However, further efforts towards devising a standard protocol are needed to enable formal validation of BALF-PCR.

## Introduction

Invasive aspergillosis (IA) is the most common opportunistic invasive fungal infection in immunocompromised patients, especially those with prolonged neutropenia [1]. In patients with hematological malignancies (HM), the prevalence of IA ranges from 1–15% and mortality can reach as high as 90%, despite the availability of several active antifungal agents [1].

Early diagnosis of IA remains a challenge, and few diagnostic methods are available. The galactomannan (GM) assay may be useful in establishing an earlier diagnosis and may result in improved outcomes for immunocompromised patients [2], [3]. The specificity of GM detection in serum generally reaches over 90%. In bronchoalveolar lavage fluid (BALF), GM detection can lead to an earlier diagnosis of IA in patients with HM, yielding an increased sensitivity when compared with serum, e.g., 85–100% versus 47% [4].

Molecular diagnostic techniques such as nucleic acid detection by polymerase chain reaction (PCR) are emerging as potentially more sensitive and rapid than conventional techniques for the diagnosis of IA [5], [6]. In addition to being helpful in IA diagnosis, DNA amplification with an integrated system for species-level identification based on melt-curve profiles or via an additional probe, would be useful to save time and refine the diagnosis of specific infections, allowing for administration of targeted antifungal therapy based on species-level identification [7]. A systematic review, which assessed the use of PCR on blood and serum samples, showed its clinical value and recommended standardization of PCR platforms [8].

BALF is routinely used to assess for the presence of fungi at the site of pulmonary infection. Conventional microbiological techniques like culture and histology of BALF are most commonly used for the diagnosis of IA, but these techniques have suboptimal sensitivity [9]. Detection of *Aspergillus* DNA in BALF yields high sensitivity and specificity. One review evaluated 15 clinical studies investigating the diagnostic efficiency of performing PCR on the BALF of IA patients and showed a promising clinical significance although with some methodological limitations [10]. Using a bivariate regression approach, we have now undertaken a systematic review of all eligible, and more recent, clinical studies to assess the accuracy of BALF-PCR as a diagnostic test for IA in immunocompromised patients.

## Materials and Methods

Two investigators searched MEDLINE and EMBASE for relevant articles published up to December 2010 for Medical Headings and text words that included the search terms “aspergillosis,” “*aspergillus*,” “polymerase chain reaction” and “bronchoalveolar lavage”. The syntax for the MEDLINE searches was as follows: “aspergillosis” OR “*aspergillus*” AND “polymerase chain reaction” AND “bronchoalveolar lavage”. The reference lists of the included studies and review articles were also checked for further relevant studies. Searches were restricted to English-language literature on human subjects only; abstracts or meeting proceedings were excluded. The results were then manually searched for eligible studies. Full-text publications concerning PCR on BALF were included if (1) they used the European Organization of the Research and Treatment of Cancer/Mycoses Study Group (EORTC/MSG) or the revised EORTC/MSG criteria, as a reference standard [11], [12], (2) for studies published before the designation of these criteria in 2002, equivalent but non-identical criteria were used as a reference standard, (3) the studies reported the data separately on true-positive, false-positive, false-negative, and true-negative results of the diagnostic tests, and (4) the studies included immunocompromised or at-risk patients. To avoid selection bias, studies with populations fewer than 10 were excluded.

Data extraction was performed independently by two reviewers and any uncertainties or disagreements were resolved by discussion. The quality of the selected studies was assessed as recommended in the Standards for the Reporting of Diagnostic Accuracy (STARD) by using 14 items of the Quality Assessment of Diagnostic Accuracy Studies (QUADAS) lists [13], [14].

### Statistical analysis

To calculate test accuracy, we defined proven and probable patients as having IA, and possible and no IA patients as not having IA, to construct two-by-two tables, according to the revised EORTC/MSG criteria [12]. We also constructed other two-by-two tables (proven IA vs probable, possible, and no IA).

By undertaking a bivariate regression approach, we calculated pooled estimates of sensitivity (SEN) and specificity (SPE) as the main outcome measures, and constructed hierarchical summary receiver operating characteristic (SROC) curves [15]. Based on random-effects models, this bivariate approach investigates potential between-study heterogeneity and incorporates the possible correlation between the SEN and the SPE. Using the pooled SEN and SPE, positive and negative likelihood ratios (PLR and NLR, respectively) were also calculated.

Heterogeneity was assessed through the test of inconsistency (*I^2^*) of the pooled diagnostic odds ratios (DORs) [16]. DOR indicated the accuracy of a diagnostic test, corresponding to particular pairings of SEN and SPE. It illustrated the odds of positive test results in participants with the disease compared with the odds of positive results in those without the disease. The mean DOR was used as an accuracy index and was performed by classic meta-analytic pooling [17].

Potential heterogeneity was explored by subgroup analyses [18], [19]. Covariates clearly reported by more than 80% of studies were analyzed: population (only HM vs. mixed/other), design (cohort vs. case-control), data collection (prospective vs. retrospective), EORTC/MSG criteria (yes vs. no), PCR method (quantitative vs. other), primer design (*A.fumigatus*-specific vs. other), cell wall disruption method (commercial vs. “in house”), and DNA extraction method (commercial vs. “in house”). Deeks's funnel plot was used to inspect publication bias [20]. Posttest probability(PTP) was calculated by using the overall prevalence of 19% with Fagan nomograms [21]. All analyses were performed using STATA, version 10 (Stata Corp., College Station, TX) with the module “Midas” [21]. All statistical tests were two sided, with *P* values less than 0.05 denoting statistical significance.

## Results

Our search criteria identified 2361 studies from literature. After screening the titles and abstracts, 101 articles were selected for full-text review and 84 studies were discarded for various reasons. In two publications with suspected overlapping data from the same medical services, we chose to include the larger of the studies [22], [23]. Ultimately, 17 studies with 1191 patients met the inclusion criteria and were included in the systematic review [23]–[39].

The characteristics of these studies are detailed in Table 1. Overall, there were 1296 clinical BALF samples from 1191 patients. Most patients with HM received either chemotherapy or a hematopoietic stem-cell transplant. The average prevalence of proven and probable IA across cohort studies was 19% (range: 5.77–47.37%), which was higher than that reported in some studies [3], [40]. The relatively higher prevalence might result from the different populations among these reviews. The participants of the included studies mainly constituted patients with HM accompanied by pulmonary infiltration.

**Table 1:** Characteristics of studies included in the analysis.

Study	Study population	Age group	Sample processing	Study design	Reference standard	Radiology-based BALF	Patients (*n*)	Proven and probable IA (*n*)
Bretagne 1995^24^	HM, HIV	NR	Prospective	Cohort	Similar^1^	NR	52	3
Buchheidt 2002^23^	allogeneic HSCT, HM	Adult	Prospective	Cohort	Similar	Y	141	26
Frealle 2009^25^	HM	Adult	Retrospective	Case-control	EORTC/MSG	NR	57	25
Hayette 2001^26^	HM, SOC, CD	NR	Retrospective	Cohort	Similar	NR	74	10
Jones 1998^27^	HM	NR	Retrospective	Case-control	Similar	NR	69	12
Khot 2008^28^	HSCT, HM, pulmonary infiltrates suggestive of pneumonia	Adult	Retrospective	Cohort	EORTC/MSG	Y	81	13
Melchers 1994^29^	HM	Adult	Retrospective	Case-control	EORTC/MSG	NR	14	6
Musher 2004^30^	HSCT	NR	Retrospective	Case-control	EORTC/MSG	NR	93	46
Raad 2002^31^	HM, SOC, pulmonary infiltrates suggestive of pneumonia	Adult	Prospective	Cohort	EORTC/MSG	NR	249	32
Rantakokko 2003^32^	HM	NR	Retrospective	Case-control	EORTC/MSG	NR	66	11
Sanguinetti 2003^33^	HM, pulmonary infiltrates suggestive of pneumonia	Adult	Retrospective	Cohort	EORTC/MSG	Y	44	20
Shahid 2008^34^	BC	Adult	Retrospective	Cohort	EORTC/MSG	Y	69	23
Skladny 1999^35^	HM	NR	Retrospective	Case-control	EORTC/MSG	Y	65	9
Spiess 2003^36^	HM, pulmonary infiltrates suggestive of pneumonia	NR	Retrospective	Case-control	EORTC/MSG	Y	31	11
Spreadbury 1993^37^	HM,SOC	Adult	Retrospective	Case-control	Similar	NR	16	3
Tang 1993^38^	HM, BMT	Adult	Retrospective	Case-control	Similar	NR	50	4
Verweij 1995^39^	HM	NR	Retrospective	Cohort	Similar	Y	19	9

1 Studies that used criteria that were similar but not identical to the EORTC/MSG criteria.

Abbreviation: BALF, bronchoalveolar lavage fluid; BC, Bronchogenic carcinoma; BMT, bone marrow transplantation; CD, corticosteroid dependent; EORTC/MSG, European Organization of the Research and Treatment of Cancer/Mycoses Study Group; HIV, human immunodeficiency virus; HM, hematological malignancy; HSCT, hematopoietic stem-cell transplant; IA, invasive aspergillosis; NR, not reported; SOC, solid-organ cancer; Y, yes.

Ten studies used the EORTC/MSG criteria, whereas seven studies used similar criteria that were not identical to the EORTC/MSG criteria. Three studies included eligible patients exhibiting persistent fever who were unresponsive to first-line broad-spectrum antibiotic treatment [25], [27], [35].

**Figure 1:**
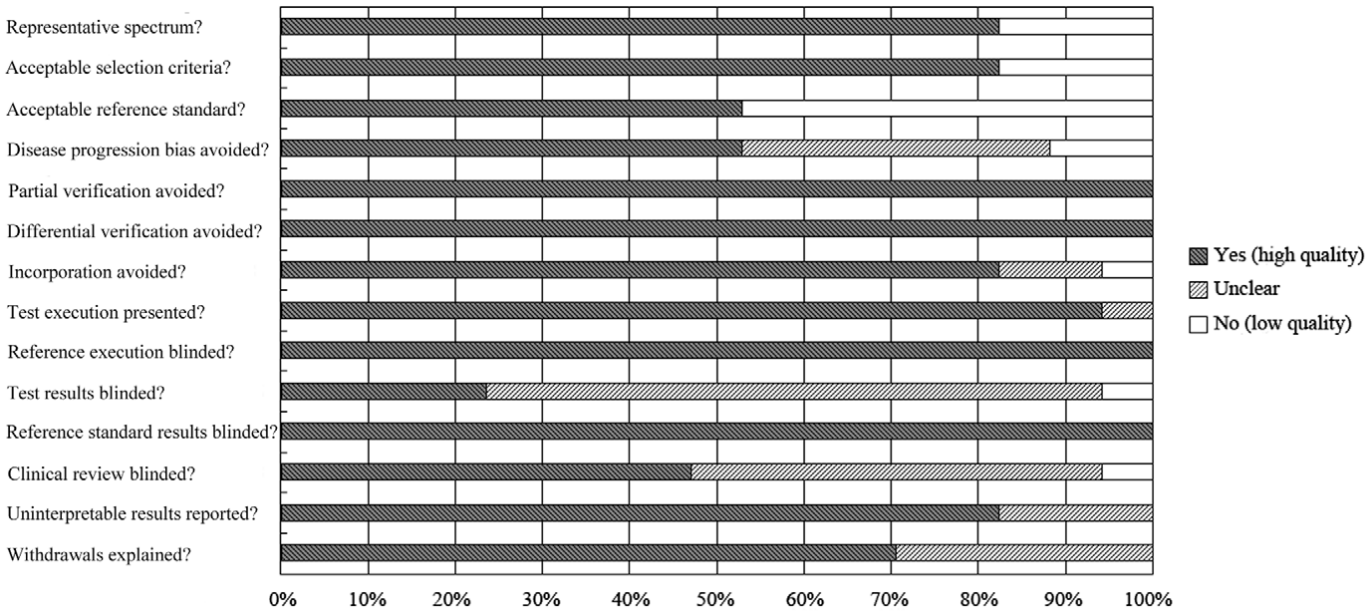
Methodological quality graph of all 17 studies. Data are presented as a percentages bar across all included studies, according to the Quality Assessment of Diagnostic Accuracy Studies lists.

The details of the PCR techniques used are summarized in Table 2. BALF was used in all studies (volume range: 0.1–5 ml). Six studies used quantitative PCR (qPCR) in *Aspergillus* DNA determination and the remainder used end-point PCR or semi-quantitative PCR. The quality of all studies was generally high, meeting on average 10 of the 14 QUADAS criteria (Fig. 1).

**Table 2:** Technical details of the PCR methods used in the included studies.

Study	BALF sample volume(ml)	Samplecentrifugation^1^	Cell wall disruption	DNA extraction methods	PCR experimental method	Primer design	Appropriate controls	
							IAC	Extraction controls^2^
Bretagne 1995^24^	1.5	NR	SDS, proteinase	Phenol-chloroform	Competitive PCR	*A. fumigatus species*	Yes	No
Buchheidt 2002^23^	1.5	Yes	Lyticase	Phenol-chloroform	Nested PCR	*Aspergillus species*	Yes	Neg
Frealle 2009^25^	0.2	Yes	QIAamp DNA Mini Kit	QIAamp	qPCR	*A. fumigatus species*	Yes	Pos/Neg
Hayette 2001^26^	0.5	No	SDS, proteinase	Phenol-chloroform	Nested PCR	*A. fumigatus and A. flavus species*	Yes	No
Jones 1998^27^	0.2	No	SDS, proteinase	Gentra pure	PCR-ELISA	*Aspergillus species*	Yes	Neg
Khot 2008^28^	2–5	Yes	MasterPure Yeast Kit	MasterPure	qPCR^3^	*Aspergillus species*	Yes	Pos/Neg
Melchers 1994^29^	NR	Yes	SDS, proteinase	Phenol-chloroform	End-point PCR	*Aspergillus species*	No	Neg
Musher 2004^30^	1.5	Yes	MasterPure Yeast kit	MasterPure	qPCR	*Aspergillus species*	Yes	Neg
Raad 2002^31^	1	Yes	SDS, proteinase	Phenol-chloroform	End-point PCR	*Aspergillus species*	Yes	No
Rantakokko 2003^32^	1.5	Yes	SDS, proteinase	Phenol-chloroform	qPCR	*A. fumigatus species*	Yes	No
Sanguinetti 2003^33^	1.5	NR	DNeasy Plant Mini Kit	DNeasy	qPCR^3^	*Aspergillus species*	No	No
Shahid 2008^34^	0.1	No	SDS, proteinase	Phenol-chloroform	End-point PCR	*A. fumigatus, A. flavus and A. niger species*	No	Neg
Skladny 1999^35^	NR	Yes	SDS, proteinase	Phenol-chloroform	Nested PCR(two step)	*Aspergillus species*	Yes	No
Spiess 2003^36^	NR	Yes	SDS, proteinase	Phenol-chloroform	qPCR and nested PCR^3,4^	*A. fumigatus species*	No	Neg
Spreadbury 1993^37^	0.25	No	SDS, proteinase	Phenol-chloroform	End-point PCR	*A. fumigatus species*	Yes	Neg
Tang 1993^38^	0.25	No	SDS, proteinase	Phenol-chloroform	End-point PCR	*A. fumigatus and A. flavus species*	No	Neg
Verweij 1995^39^	NR	Yes	SDS, proteinase	Phenol-chloroform	End-point PCR	*A. fumigatus species*	No	Neg

1 Studies that used a BALF pellet for detection after centrifugation. ^2^Positive or negative extraction control. ^3^Studies that quantified the fungal load in the BALF. ^4^Study that used two PCR methods in the same population. Only the qPCR data are included.

Abbreviations: BALF, bronchoalveolar lavage fluid; ELISA, enzyme-linked immunosorbent assay; IAC, internal amplification control; Neg, negative; NR, not reported; PCR, polymerase chain reaction; Pos, positive; qPCR, quantitative real-time PCR; SDS, sodium dodecyl sulphate buffer.

### Data synthesis and metaanalysis

For all the studies, the pooled DOR was 122 (95% confidence interval (CI) 41–363). Table 3 shows the SEN, SPE, PLR, NLR, and DOR. The wide range of SEN was mainly because of the variance of IA cases in different studies. We found substantial heterogeneity among studies for all modalities because all I^2^ values were above 50%.

**Table 3:** Synthesized statistics with two definition criteria.

Patient definition	Studies(*n*)	DOR	Pooled SEN	Pooled SPE	PLR	NLR
Proven and probable versus possible and no IA	17	122 (41–363)^1^	0.91 (0.79–0.96)	0.92 (0.87–0.96)	11.9 (6.8–20.8)	0.10 (0.04–0.24)
Proven versus probable, possible and no IA	8	49 (17–140)	0.90 (0.77–0.96)	0.84 (0.81–0.87)	5.7 (4.6–7.0)	0.12 (0.05–0.29)

1 Values in parentheses are 95% confidence intervals.

Abbreviations: DOR, diagnostic odds ratio; IA, invasive aspergillosis; NLR, negative likelihood ratio; PLR, positive likelihood ratio; SEN, sensitivity; SPE, specificity.

**Figure 2:**
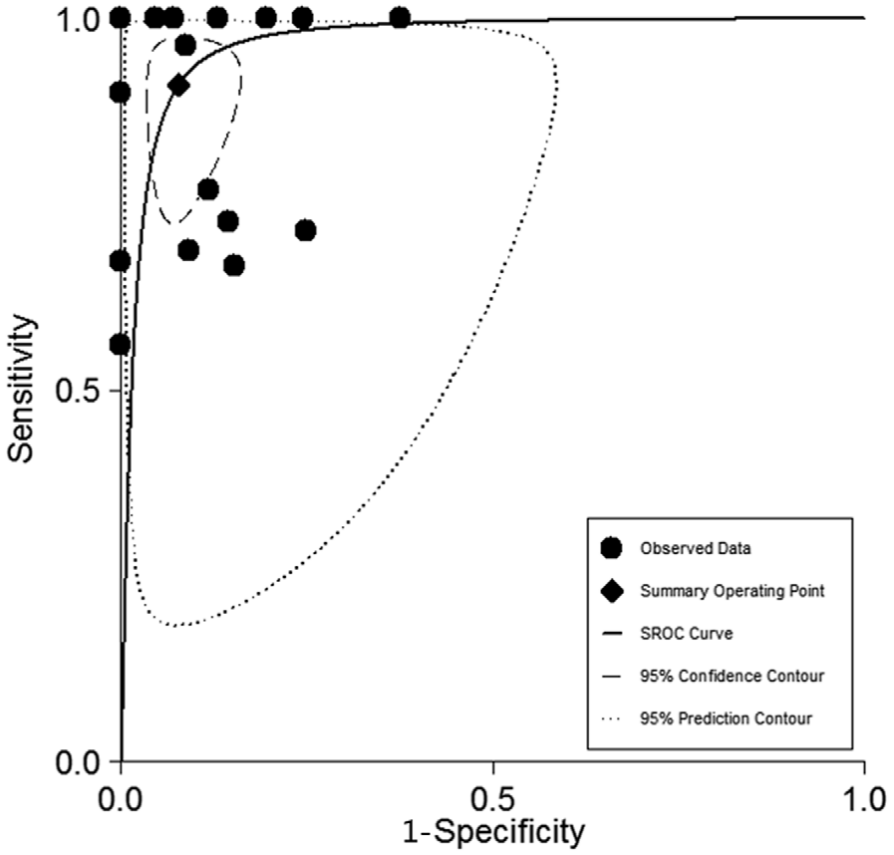
SROC curves from the bivariate model for proven and probable cases. The region enclosed by the dashed line (confidence contour) contains likely combinations of the mean value of sensitivity and specificity. The region enclosed by the dotted line (prediction contour) demonstrates more uncertainty as to where the likely values of sensitivity and specificity might occur for individual studies. SROC, summary receiver operating characteristic.

The SROC curve represents the relationship between SEN and SPE across studies, determining the presence of a threshold effect. Based on the bivariate approach which estimates not only the strength but also the shape of the correlation between SEN and SPE, a 95% confidence ellipse and a 95% prediction ellipse were drawn(Fig. 2). The area under the SROC curve (AUC) was 0.97 (95% CI 0.95–0.98), signifying the high discriminatory ability of BALF–PCR.

**Figure 3:**
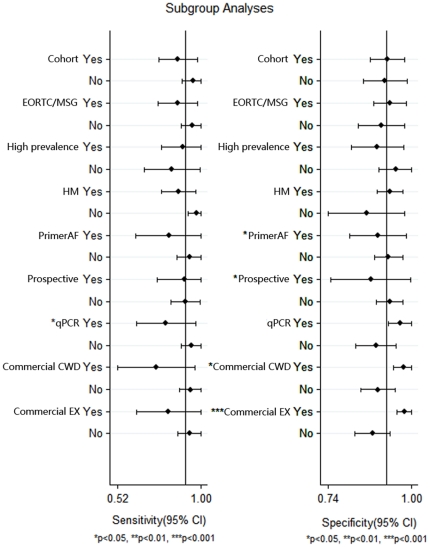
Forest plot of subgroup analyses for sensitivity and specificity. EORTC/MSG, similar to the European Organization of the Research and Treatment of Cancer/Mycoses Study Group (EORTC/MSG) criteria 2008; Higher prevalence, prevalence above 23%; HM, hematologic malignancy; Primer AF, *A.fumigatus*-specific primer; Commercial CWD, commercial kit for cell wall disruption; Commercial EX, commercial kit for DNA extraction; * *P*<0.05; ** *P*<0.01; *** *P*<0.001.

Subgroup analyses showed that the SEN of qPCR was significantly lower than that of other types of PCR (Fig. 3). Studies using commercial kits for cell wall disruption and DNA extraction achieved significantly higher SPE than those using an “in house” method. These covariates did not affect the SEN yet. We also found that using *A.fumigatus* species-specific primers led to a significantly lower SPE than did using other primers (mostly genus-specific primers).

**Figure 4:**
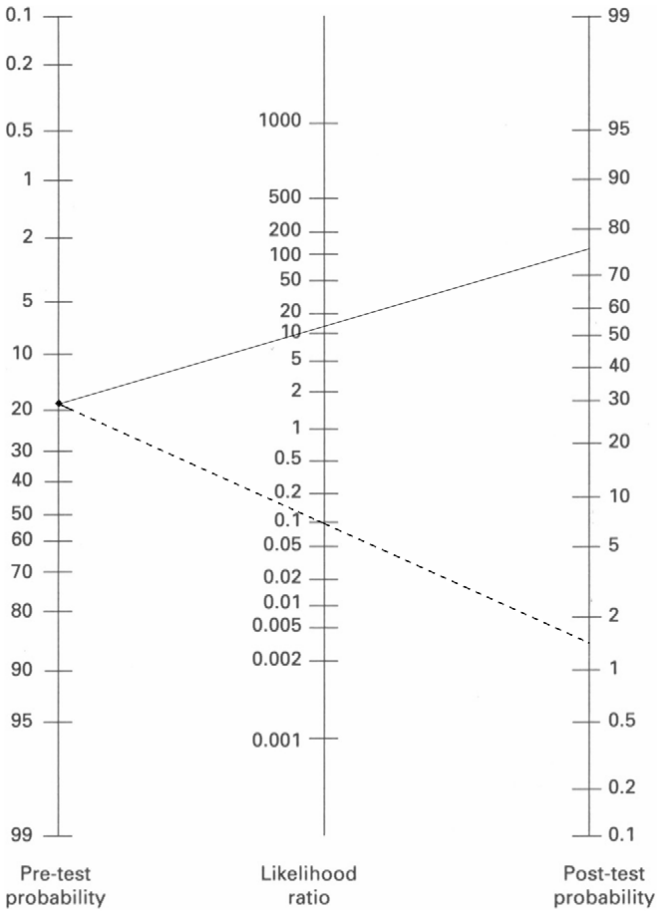
Fagan's nomogram for calculating posttest probabilities (PTPs). A straight edge was used to link the pretest probability of IA with the PTP, by crossing the likelihood ratio line at a point that describes the results obtained. The solid line extends from the prevalence to the PLR; the dotted line extends from the prevalence to the NLR.

Non-publication bias was detected by the Deek's funnel plot asymmetry test (*P* = 0.55) [19]. The nomogram of Fagan demonstrated that the PCR assay increased the probability of IA nearly five-fold when the results were positive, and decreased the probability to 1.5% when the results were negative (Fig. 4)[21].

## Discussion

The frequency of IA has increased with the increasing number of high-risk patients. The most common site of infection is pulmonary and IA can easily disseminate to other organs [41]. Clinically, the gold standard for the diagnosis of IA still requires an invasive procedure to provide histopathologic or cytopathologic evidence. Unfortunately, the patient's status often prohibits the use of invasive techniques. Culturing of the causative agent can result in false negative or false positive results. Bronchoscopy with BALF seems to be a feasible diagnostic tool, with a high yield and low complication rates [42]–[44].

Serum GM detection and PCR assays seem to have diagnostic potential. Clinical trials have shown that GM assays on serum or BALF and PCR assays on serum have variable performance in different at-risk patient populations. The metaanalyses of Pfeiffer st al. and Leeflang et al. both drew similar conclusion that serum GM detection was moderately useful for diagnosing IA although there were some methodological limitations in these studies [3], [40]. In Leeflang et al.'s study, in which only proven and probable cases were considered, the overall SEN increased by 17% (from 62% to 79%) with a decrease in the threshold from 1.5 to 0.5, and the overall SPE decreased by 13% (from 95% to 82%). Mengoli et al. determined the SEN and SPE of PCR for two consecutive positive samples to be 0.75 and 0.87, respectively, and concluded that two positive tests are required to confirm the diagnosis, whereas a single PCR-negative result is sufficient to exclude a diagnosis of IA [8]. Wang et al.'s found that, as a diagnostic tool, the BALF-GM assay was better than serum GM detection [45]. This study showed that the BALF-GM assay had a SEN of 0.90 and a SPE of 0.94 for proven and probable IA, which were both higher than the corresponding values for the serum GM assay.

We undertook the present metaanalysis aiming to establish the overall accuracy of the BALF-PCR assay for the diagnosis of IA and to help standardize PCR assay and normalize clinical performance. Our primary finding was that the BALF-PCR assay is a powerful tool for the diagnosis of IA in patients with hematological malignancy, and that the use of particular procedures can improve diagnostic accuracy. However, the increased SEN and SPE could be related to the high sensitivity of PCR, and possible existing fungal colonization in the bronchial tree of high-risk cases might contribute to the elevated SEN. Comparing the diagnostic tools examined in all former metaanalyses, the BALF-PCR assay had the highest area under the SROC curve (0.97) which meant that this assay had the best discriminative ability.

We also investigated likelihood ratios, which take into account the interaction between the SEN and the SPE in their calculation and describe the discriminatory properties of positive and negative test results. PLR >10 and NLR <0.1 are usually considered convincing evidence to rule in or rule out diagnoses, respectively [46]. Although three metaanalyses did not report these results [3], [8], [40], the conclusions of these studies suggested that serum GM and serum PCR could not effectively discriminate IA. In our metaanalysis, for proven and probable cases, the PLR and NLR both exceeded the threshold index and generated large and often conclusive shifts from pre-test to PTP.

It is an important objective of a metaanalysis to explore the likely causes of heterogeneity [18], [19]. In our subgroup analyses, we identified several characteristics that might account for the observed heterogeneity. The significantly lower SEN of qPCR might be due to the use of a modified PCR assay for improvement of SEN, such as nested PCR. Nested PCR formats have been widely used for *Aspergillus* in an attempt to optimize analytical sensitivity, but the requirement to open the reaction tubes means that there is considerable risk of contamination and the subsequent generation of false-positive results [47]. Recently, the real-time quantitative format has become dominant in PCR-based diagnostic studies of fungal infections [7]. The limit of its sensitivity was found to be five copies of *Aspergillus* DNA per milliliter; this is comparable to that of the commonly used nested PCR assay [48]. In addition, quantification of the fungal burden by real-time PCR may be helpful to distinguish between colonization and infection, and could possibly allow therapeutic monitoring [32]. Nowadays, real-time qPCR is the best PCR format for clinical diagnostic application.

There is a multitude of fungal DNA extraction techniques. The selected extraction method represents a compromise between efficiency, freedom from exogenous contamination, and applicability to routine high-throughput testing. The efficiency of extraction of fungal DNA may vary considerably among commercial kits [49], And this can hinder comparisons among studies. We found that studies that used commercial kits for cell wall disruption and DNA extraction achieved significantly higher SPE than those that used “in-house” methods. The uniform commercial kit for the GM assay contributes to its stable operation and comprehensive use, and we believe that a uniform PCR assay system may also promote the application of qPCR.

When designing primers for clinical diagnostic purposes, the detection of a broad range of fungi is important, as is the ability to increase SEN and ultimately identify the specific pathogen [50]. In our study, we found that using *A.fumigatus* species-specific primers led to a significantly lower SPE.of the use of narrow-spectrum primers that does not lead to a lower SPE could be caused by poor primer design. Some primers, which were designed based on sequencing data that was not comprehensively validated, have shown complementarity to the genomes of several other microorganisms, such as *Candida glabrata* and *Aspergillus oryzae* [24], [32]. During primer design, it is necessary to conduct a thorough in silico analysis based on multiple alignment of validated sequences from various databases of as many *Aspergillus* sequences as possible, plus human sequences and those of closely related fungal pathogens such as *Penicillium* and *Candida*. Since several species of *Aspergillus* are human pathogens, *A.fumigatus* species-specific primers may lower the accuracy of PCR assays. We recommend choosing *Aspergillus* genus-specific primers, to improve PCR assay accuracy. The optimal approach, in this regard, involves the application of genus-specific primers with post-amplification analysis for species determination. Genus-specific primers are directed toward conserved regions, usually within multicopy genes, which flank sequences containing species specific polymorphisms that can be exploited in post-amplification analysis [47]. However, *Aspergillus* genus-specific primers might lead to a new problem. *Penicillium*, a genus phylogenetically close to *Aspergillus*, has a high likelihood of cross reactivity within a PCR assay, which might increase the false positive rate [6]. All primers should be validated by a thorough in silico analysis using multiple databases.

Another potential problem with PCR is sample contamination from airborne particles. Fungal spores are ubiquitous in the environment, and may cause false positive results in PCR. Accordingly, measures to reduce exogenous fungal contamination are critical [7]. A laminar flow hood in an independent laboratory should be used exclusively for DNA extraction and pre-PCR processing. Other measures to reduce contamination are a unidirectional workflow pattern (pre- to post-PCR), physically separating the laboratories for pre- and post-PCR analysis, and using aerosol-resistant pipette tips and laminar flow hoods. In addition, using the uracil-DNA glycosylase enzyme and dUTP instead of dTTP in the PCR master mix can eliminate this problem by destroying amplicons prior to PCR [7]. Moreover, controls can be used to rigorously monitor contamination. Negative control reactions, comprising all the PCR reagents except the template DNA, are essential, and can be introduced at any point in theassay, such as at sample acquisition, handling, storage, and DNA extraction.

It is still difficult for BALF-PCR to distinguish colonization from invasive infection. More importantly, the isolation of *Aspergillus* from the respiratory tract should arouse vigilance, especially in high-risk patients. The isolation of *Aspergillus* from the respiratory tract may represent one of three scenarios: (1) evidence of current disease, (2) true colonization, or (3) a marker for the probable development of invasive disease. A previous study demonstrated that a positive PCR result from BALF at the time of bone marrow transplant conditioning was predictive of the subsequent development of invasive pulmonary aspergillosis [51].

The PCR assay, especially qPCR, is becoming popular in the clinical diagnosis “toolbox”, such as for *Pneumocystis* pneumonia diagnosis [52]. As the operation of PCR becomes more automated, and extraction methods and targets become commercially available, this tool should play an ever-greater role. However, because at present most laboratories perform in-house PCR assays, extensive validation and standardization is requied. An initiative is currently in progress, to devise a standard for *Aspergillus* PCR screening [8]. Once this has been achieved, formal validation shoule be possible. As long as these standardized assays are unavailable, a combination of various methods is still needed to improve the accuracy of IA diagnosis of IA.

Our study had some limitations. First, we acknowledge that the overall number of patients included in our review was relatively small. Although we aimed to incorporate all available relevact data, it is hard to ensure that no data were missed, especially unpublished data. Second, we might have introduced bias by exclusing non-English-language studies and studies with population fewer than 10. To test the latter, we reanalyzed the data including these small studies and obtained similar overall results. Third, we used the revised EORTC/MSG criteria as a reference standard which is widely accepted but not the “gold standard” for diagnosis of every patient, especially for probable IA. The disease group could be expanded when defining probable IA.

## References

[1] Walsh TJ, Anaissie EJ, Denning DW, Herbrecht R, Kontoyiannis DP (2008). Treatment of aspergillosis: clinical practice guidelines of the Infectious Diseases Society of America.. Clin Infect Dis.

[2] Pfeiffer CD, Fine JP, Safdar N (2006). Diagnosis of invasive aspergillosis using a galactomannan assay: a meta-analysis.. Clin Infect Dis.

[3] Leeflang MM, Debets-Ossenkopp YJ, Visser CE, Scholten RJ, Hooft L (2008). Galactomannan detection for invasive aspergillosis in immunocompromized patients..

[4] Becker MJ, Lugtenburg EJ, Cornelissen JJ, Van Der Schee C, Hoogsteden HC (2003). Galactomannan detection in computerized tomography-based broncho-alveolar lavage fluid and serum in haematological patients at risk for invasive pulmonary aspergillosis.. Br J Haematol.

[5] Donnelly JP (2006). Polymerase chain reaction for diagnosing invasive aspergillosis: getting closer but still a ways to go.. Clin Infect Dis.

[6] Hope WW, Walsh TJ, Denning DW (2005). Laboratory diagnosis of invasive aspergillosis.. Lancet Infect Dis.

[7] Khot PD, Fredricks DN (2009). PCR-based diagnosis of human fungal infections.. Expert Rev Anti Infect Ther.

[8] Mengoli C, Cruciani M, Barnes RA, Loeffler J, Donnelly JP (2009). Use of PCR for diagnosis of invasive aspergillosis: systematic review and meta-analysis.. Lancet Infect Dis.

[9] Reichenberger F, Habicht J, Matt P, Frei R, Soler M (1999). Diagnostic yield of bronchoscopy in histologically proven invasive pulmonary aspergillosis.. Bone Marrow Transplant.

[10] Tuon FF (2007). A systematic literature review on the diagnosis of invasive aspergillosis using polymerase chain reaction (PCR) from bronchoalveolar lavage clinical samples.. Rev Iberoam Micol.

[11] Ascioglu S, Rex JH, de Pauw B, Bennett JE, Bille J (2002). Defining opportunistic invasive fungal infections in immunocompromised patients with cancer and hematopoietic stem cell transplants: an international consensus.. Clin Infect Dis.

[12] De Pauw B, Walsh TJ, Donnelly JP, Stevens DA, Edwards JE (2008). Revised definitions of invasive fungal disease from the European Organization for Research and Treatment of Cancer/Invasive Fungal Infections Cooperative Group and the National Institute of Allergy and Infectious Diseases Mycoses Study Group (EORTC/MSG) Consensus Group.. Clin Infect Dis.

[13] Bossuyt PM, Reitsma JB, Bruns DE, Gatsonis CA, Glasziou PP (2003). Towards complete and accurate reporting of studies of diagnostic accuracy: The STARD Initiative.. Ann Intern Med.

[14] Whiting P, Rutjes AW, Dinnes J, Reitsma J, Bossuyt PM (2004). Development and validation of methods for assessing the quality of diagnostic accuracy studies.. Health Technol Assess.

[15] Reitsma JB, Glas AS, Rutjes AW, Scholten RJ, Bossuyt PM (2005). Bivariate analysis of sensitivity and specificity produces informative summary measures in diagnostic reviews.. J Clin Epidemiol.

[16] Higgins JP, Thompson SG, Deeks JJ, Altman DG (2003). Measuring inconsistency in meta-analyses.. BMJ.

[17] Deeks JJ (2001). Systematic reviews in health care: Systematic reviews of evaluations of diagnostic and screening tests.. BMJ.

[18] Lijmer JG, Bossuyt PM, Heisterkamp SH (2002). Exploring sources of heterogeneity in systematic reviews of diagnostic tests.. Stat Med.

[19] Petitti DB (2001). Approaches to heterogeneity in meta-analysis.. Stat Med.

[20] Deeks JJ, Macaskill P, Irwig L (2005). The performance of tests of publication bias and other sample size effects in systematic reviews of diagnostic test accuracy was assessed.. J Clin Epidemiol.

[21] Fagan TJ (1975). Letter: Nomogram for Bayes theorem.. N Engl J Med.

[22] Buchheidt D, Baust C, Skladny H, Ritter J, Suedhoff T (2001). Detection of Aspergillus species in blood and bronchoalveolar lavage samples from immunocompromised patients by means of 2-step polymerase chain reaction: clinical results.. Clin Infect Dis.

[23] Buchheidt D, Baust C, Skladny H, Baldus M, Brauninger S (2002). Clinical evaluation of a polymerase chain reaction assay to detect Aspergillus species in bronchoalveolar lavage samples of neutropenic patients.. Br J Haematol.

[24] Bretagne S, Costa JM, Marmorat-Khuong A, Poron F, Cordonnier C (1995). Detection of Aspergillus species DNA in bronchoalveolar lavage samples by competitive PCR.. J Clin Microbiol.

[25] Frealle E, Decrucq K, Botterel F, Bouchindhomme B, Camus D (2009). Diagnosis of invasive aspergillosis using bronchoalveolar lavage in haematology patients: influence of bronchoalveolar lavage human DNA content on real-time PCR performance.. Eur J Clin Microbiol Infect Dis.

[26] Hayette MP, Vaira D, Susin F, Boland P, Christiaens G (2001). Detection of Aspergillus species DNA by PCR in bronchoalveolar lavage fluid.. J Clin Microbiol.

[27] Jones ME, Fox AJ, Barnes AJ, Oppenheim BA, Balagopal P (1998). PCR-ELISA for the early diagnosis of invasive pulmonary aspergillus infection in neutropenic patients.. J Clin Pathol.

[28] Khot PD, Ko DL, Hackman RC, Fredricks DN (2008). Development and optimization of quantitative PCR for the diagnosis of invasive aspergillosis with bronchoalveolar lavage fluid.. BMC Infect Dis.

[29] Melchers WJ, Verweij PE, den Hurk P v, van BA, De Pauw BE (1994). General primer-mediated PCR for detection of Aspergillus species.. J Clin Microbiol.

[30] Musher B, Fredricks D, Leisenring W, Balajee SA, Smith C (2004). Aspergillus galactomannan enzyme immunoassay and quantitative PCR for diagnosis of invasive aspergillosis with bronchoalveolar lavage fluid.. J Clin Microbiol.

[31] Raad I, Hanna H, Huaringa A, Sumoza D, Hachem R (2002). Diagnosis of invasive pulmonary aspergillosis using polymerase chain reaction-based detection of aspergillus in BAL.. Chest.

[32] Rantakokko-Jalava K, Laaksonen S, Issakainen J, Vauras J, Nikoskelainen J (2003). Semiquantitative detection by real-time PCR of Aspergillus fumigatus in bronchoalveolar lavage fluids and tissue biopsy specimens from patients with invasive aspergillosis.. J Clin Microbiol.

[33] Sanguinetti M, Posteraro B, Pagano L, Pagliari G, Fianchi L (2003). Comparison of real-time PCR, conventional PCR, and galactomannan antigen detection by enzyme-linked immunosorbent assay using bronchoalveolar lavage fluid samples from hematology patients for diagnosis of invasive pulmonary aspergillosis.. J Clin Microbiol.

[34] Shahid M, Malik A, Bhargava R (2008). Bronchogenic carcinoma and secondary aspergillosis--common yet unexplored: evaluation of the role of bronchoalveolar lavage-polymerase chain reaction and some nonvalidated serologic methods to establish early diagnosis.. Cancer.

[35] Skladny H, Buchheidt D, Baust C, Krieg-Schneider F, Seifarth W (1999). Specific detection of Aspergillus species in blood and bronchoalveolar lavage samples of immunocompromised patients by two-step PCR.. J Clin Microbiol.

[36] Spiess B, Buchheidt D, Baust C, Skladny H, Seifarth W (2003). Development of a LightCycler PCR assay for detection and quantification of Aspergillus fumigatus DNA in clinical samples from neutropenic patients.. J Clin Microbiol.

[37] Spreadbury C, Holden D, Aufauvre-Brown A, Bainbridge B, Cohen J (1993). Detection of Aspergillus fumigatus by polymerase chain reaction.. J Clin Microbiol.

[38] Tang CM, Holden DW, Aufauvre-Brown A, Cohen J (1993). The detection of Aspergillus spp. by the polymerase chain reaction and its evaluation in bronchoalveolar lavage fluid.. Am Rev Respir Dis.

[39] Verweij PE, Latge JP, Rijs AJ, Melchers WJ, De Pauw BE (1995). Comparison of antigen detection and PCR assay using bronchoalveolar lavage fluid for diagnosing invasive pulmonary aspergillosis in patients receiving treatment for hematological malignancies.. J Clin Microbiol.

[40] Pfeiffer CD, Fine JP, Safdar N (2006). Diagnosis of invasive aspergillosis using a galactomannan assay: a meta-analysis.. Clin Infect Dis.

[41] Rapp RP (2004). Changing strategies for the management of invasive fungal infections.. Pharmacotherapy.

[42] Hummel M, Rudert S, Hof H, Hehlmann R, Buchheidt D (2008). Diagnostic yield of bronchoscopy with bronchoalveolar lavage in febrile patients with hematologic malignancies and pulmonary infiltrates.. Ann Hematol.

[43] Kuehnhardt D, Hannemann M, Schmidt B, Heider U, Possinger K (2009). Therapeutic implication of BAL in patients with neutropenia.. Ann Hematol.

[44] Hohenthal U, Itala M, Salonen J, Sipila J, Rantakokko-Jalava K (2005). Bronchoalveolar lavage in immunocompromised patients with haematological malignancy--value of new microbiological methods.. Eur J Haematol.

[45] Guo YL, Chen YQ, Wang K, Qin SM, Wu C (2010). Accuracy of BAL galactomannan in diagnosing invasive aspergillosis: a bivariate metaanalysis and systematic review.. Chest.

[46] Jaeschke R, Guyatt GH, Sackett DL (1994). Users' guides to the medical literature. III. How to use an article about a diagnostic test. B. What are the results and will they help me in caring for my patients? The Evidence-Based Medicine Working Group.. JAMA.

[47] Hope WW, Walsh TJ, Denning DW (2005). Laboratory diagnosis of invasive aspergillosis.. Lancet Infect Dis.

[48] Bolehovska R, Pliskova L, Buchta V, Cerman J, Hamal P (2006). Detection of Aspergillus spp. in biological samples by real-time PCR.. Biomed Pap Med Fac Univ Palacky Olomouc Czech Repub.

[49] Loeffler J, Hebart H, Bialek R, Hagmeyer L, Schmidt D (1999). Contaminations occurring in fungal PCR assays.. J Clin Microbiol.

[50] Imhof A, Schaer C, Schoedon G, Schaer DJ, Walter RB (2003). Rapid detection of pathogenic fungi from clinical specimens using LightCycler real-time fluorescence PCR.. Eur J Clin Microbiol Infect Dis.

[51] Einsele H, Quabeck K, Muller KD, Hebart H, Rothenhofer I (1998). Prediction of invasive pulmonary aspergillosis from colonisation of lower respiratory tract before marrow transplantation.. Lancet.

[52] Huggett JF, Taylor MS, Kocjan G, Evans HE, Morris-Jones S (2008). Development and evaluation of a real-time PCR assay for detection of Pneumocystis jirovecii DNA in bronchoalveolar lavage fluid of HIV-infected patients.. Thorax.

